# Exploring public perceptions of the public good use of data for research and statistics

**DOI:** 10.23889/ijpds.v8i2.2239

**Published:** 2023-09-14

**Authors:** Shayda Kashef, Sofi Nickson, Mary Cowan

**Affiliations:** 1 ADR UK, London, United Kingdom; 2 Office for Statistics Regulation, Newport, United Kingdom; 3 Office for Statistics Regulation, London, United Kingdom

## Objectives

Sharing and use of data for research and statistics underpins creation of better evidence for decision-making. For public data to be shared among organisations, the research or statistics created with the data must serve the public good. We argue decisions about what public good means should involve the public.

## Method

We carried out a public dialogue to better understand public perceptions of the ‘public good’ use of data for research and statistics. This included deliberative in-person and online workshops with 68 people in Belfast, Cardiff, Glasgow, and London. The participant demographics were purposefully diverse with little to no formal knowledge of data or statistics. Through discussions in small groups, and knowledge exchange with data and statistics professionals, participants had the opportunity to hear more about statistics and data sharing and linkage, and could share their insights into this work from a personal perspective.

## Results

24 hours of deliberative discussions were analysed and distilled into five main findings, which demonstrate that participants want the public to have a say in how the public good is defined in the context of decision-making on data sharing and use. The participants also strongly expressed that data for research and statistics should address inequalities in society and minimise harms; both of which could be partly achieved through meaningful public engagement. Participants also suggested that they would like to see more communication around the benefits of the use of data for research and statistics. Finally, they were broadly supportive of more data sharing under best practice safeguards, over and above existing legal frameworks.

## Conclusion

Insights from deliberative discussions with members of the public on their perceptions of the public good use and sharing of data is reciprocally beneficial. It demonstrates trustworthiness to the public by meaningfully involving them in decisions about their data and ensures organisations can receive feedback on how to maximise the public good of their work.

